# ANNOUNCEMENTS & RESOURCES

**Published:** 2017-03-03

## IAPB Vision Atlas

**Figure:**
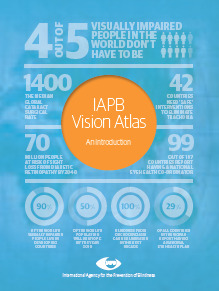


The IAPB Vision Atlas website isa compilation of the latest data and evidence relevant to all those who believe that in the 21st Century no one should have to live with avoidable blindness or sight loss - from eye conditions many of which can be easily treated or prevented and for which cost-effective solutions are readily available.

The IAPB Vision Atlas is designed around two main sets of data: the estimates of the burden of blindness and visual impairment made by the Vision Loss Expert Group (VLEG) and national level performance against the key indicators laid out in the World Health Assembly resolution 66.4 ‘Universal Eye Health: a Global Action Plan 2014 – 2019’ (the GAP).

Do visit it here: **http://atlas.iapb.org**

## Courses

### MSc Public Health for Eye Care, London School of Hygiene & Tropical Medicine

10 fully funded scholarships available for Commonwealth Country Nationals. Course aims to provide eye health professionals with the public health knowledge and skills required to reduce blindness and visual disability in their setting. For more information visit: **www.lshtm.ac.uk/study/masters/mscphec.html** or email **Romulo.Fabunan@Lshtm.ac.uk**

**Eye Banking Course:** New international qualification course for eye bankers. Suitable to all service and experience levels. The *Specialist Certificate* course starts in September 2017 (with option to work towards *Graduate Certificate* afterwards). Expressions of Interest via: **http://commercial.unimelb.edu.au/custom-education/courses/eyebankingsc** or please contact Heather Machin, Subject Coordinator, via: **heather.machin@unimelb.edu.au**

### University of Cape Town Community Eye Health Institute

**www.health.uct.ac.za** or email **chevron.vanderross@uct.ac.za**

### Kilimanjaro Centre for Community Ophthalmology International

**www.kcco.net** or contact Genes Mng'anga at **genes@kcco.net**

### Lions Medical Training Centre

Write to the Training Coordinator, Lions Medical Training Centre, Lions SightFirst Eye Hospital, PO Box 66576-00800, Nairobi, Kenya.

Tel: +254 20 418 32 39

## Subscriptions

Contact Anita Shah **admin@cehjournal.org**

### Subscribe to our mailing list

**web@cehjournal.org** or visit **www.cehjournal.org/subscribe**

### Visit us online

**www.cehjournal.org www.facebook.com/CEHJournal https://twitter.com/CEHJournal**

